# Effects of repeated sprint training with constant or incremental rest intervals on the decrement score, blood lactate concentration, and Wingate anaerobic test performance: a pilot study

**DOI:** 10.3389/fspor.2025.1641788

**Published:** 2025-11-10

**Authors:** Nobuyasu Tomabechi, Sho Nakazawa, Takayuki Okuhara, Ryo Aoki

**Affiliations:** 1 Liberal Arts and Sciences, Hokkaido University of Sciences, Sapporo, Hokkaido, Japan; 2 Sports Training Center, Nippon Sport Science University, Setagaya, Tokyo, Japan; 3 High Performance Center, Nippon Sport Science University, Setagaya, Tokyo, Japan; 4 Faculty of Sport Science, Nippon Sport Science University, Setagaya, Tokyo, Japan; 5 Faculty of Economics, North Asia University, Akita, Akita, Japan; 6 Shimizu Corporation Koutoh Blue Sharks, Yokohama, Kanagawa, Japan; 7 Meiji Yasuda Health Promotion Center, Meiji Yasuda Health Development Foundation, Tokyo, Japan; 8 Well-I Inc., Shinagawa, Tokyo, Japan

**Keywords:** muscle fatigue, glycolytic metabolism, maximal pedaling, peak power, mean power

## Abstract

The effects of repeated sprint training with constant rest intervals (CRIs) vs. incremental rest intervals (IRIs), without matching the total rest duration, on muscle fatigue, glycolytic metabolism, and anaerobic work capacity remain unclear. Therefore, we aimed to examine the effects of repeated sprint training with CRIs vs. IRIs on the decrement score, a measure of muscle fatigue; blood lactate concentration; and Wingate anaerobic test performance. We hypothesized that the decrement score, a measure of muscle fatigue, would be significantly lower after repeated sprint training with IRIs than after repeated sprint training with CRIs. Furthermore, we hypothesized that the blood lactate concentration would be equivalent with IRIs or CRIs and that the Wingate anaerobic test performance would be equally improved. In total, 17 male participants performed nine sessions of repeated sprint training (10 sets × 10-s maximal pedaling) within 3 weeks. They were assigned to either the group that underwent repeated sprint training with CRIs (30-s rest intervals between sets) or the same training with IRIs (30-s rest intervals until the fifth set, and then the rest interval was increased by 10-s for each set from the sixth set onwards). The decrement score showed no statistically significant difference between the groups in all sessions. The blood lactate concentration was measured in sessions one and nine and significantly decreased from sessions one to nine in both groups (*p* < 0.05). However, the blood lactate concentration did not differ significantly between the groups in sessions one and nine. From pre-training to post-training, the peak power during the Wingate anaerobic test did not increase in either group. The mean power during the Wingate anaerobic test increased significantly in both groups (*p* < 0.05), but there was no significant difference between the groups. These results suggest that RST with IRIs may not provide additional benefits compared to RST with CRIs in untrained young adults.

## 1 Introduction

Repeated sprint training (RST) is characterized by repeating short-duration sprints (≤10-s) interspersed with brief recovery periods (≤60-s) and is an efficient training method for team and racket sport athletes who require an excellent repeated-sprint ability (1, 2). Due to the high contribution of anaerobic metabolism, RST is commonly used to enhance anaerobic work capacity (2). The rest interval during RST is a variable that affects physiological responses and has chronic effects on anaerobic work capacity (3–6). Therefore, it is important to elucidate the most effective rest interval methods for RST.

RST with incremental rest intervals (IRIs) adversely affects sprint performance when matching the total rest duration. In numerous previous studies on RST, constant rest intervals (CRIs), in which the rest duration between each sprint remains the same throughout the RST, have been used and are the standard approach to arranging rest intervals in RST (3–6). However, Billaut and Basset (7) conducted the only study to date that examined repeated sprint exercises with IRIs. They reported that, when the total rest duration was matched, repeated sprint exercise with IRIs (10–50 s of recovery, increasing by 5 s at each interval) significantly lowered the total work volume compared with repeated sprint exercise with CRIs (30 s of recovery between each sprint). This adverse effect is due to the extremely small ratio of sprint duration to rest duration, namely, the work-to-rest ratio, in the first half of the repeated sprint exercise with IRIs (e.g., a work-to-rest ratio of approximately 1:1.7) (7). Hence, to clarify the effectiveness of the IRIs without underestimating it, the total rest duration during the RST should not be matched.

An increase in muscle fatigue, reflected as a decline in power output, may be controlled by using IRIs without matching the total rest duration during RST. Repeated sprint exercise with shorter rest intervals typically leads to greater muscle fatigue and reduced power output (3, 5, 8). Thus, incrementally increasing rest may help mitigate this decline. Despite this, the extent to which IRIs and CRIs without matching total rest duration differ in terms of muscle fatigue remains unclear. Muscle fatigue during RST is most validly assessed using the decrement score (S_dec_), which is calculated from the peak power across all sets (2). Therefore, a comparison of muscle fatigue when using CRIs and IRIs should be conducted using S_dec_.

It has also been speculated that there is no significant difference in blood lactate concentration (bLa), an indirect marker of increased glycolytic metabolism, when using CRIs or IRIs, and that anaerobic work capacity is equally improved by performing RST with CRIs or IRIs, without matching the total rest duration. Previous studies have not reached a consensus on whether repeated sprint exercise with a shorter rest interval is associated with a higher bLa compared to repeated sprint exercise with a longer rest interval due to different protocols being used (e.g., sprint duration, work-to-rest ratio) (3, 4, 6). However, the contribution of glycolytic metabolism is high in the first half but low in the latter half of repeated sprint exercise (2, 9). Therefore, it is possible that even if the rest interval is gradually increased in the latter half of RST, one’s glycolytic metabolism and anaerobic work capacity would be equally increased. However, the effects of RST with CRIs vs. IRIs, without matching the total rest duration, on glycolytic metabolism and anaerobic work capacity remain unclear.

Hence, this study aimed to examine the effects of 3-week RST with CRIs or IRIs on the S_dec_, bLa, and Wingate anaerobic test performance (WAnT) to determine the relative effectiveness of each protocol. We hypothesized that S_dec_ would be significantly lower after RST with IRIs than after RST with CRIs. Additionally, we hypothesized that bLa would be equivalent in RST with IRIs or CRIs and that WAnT performance would be equally improved.

## 2 Materials and methods

### 2.1 Ethics statements

Written informed consent was obtained from each participant prior to the experiment after explaining the purpose, method, and dangers of the study. This study was approved by the Ethics Committee of the Nippon Sport Science University (approval number: 021-H014) and conducted in accordance with the tenets of the Declaration of Helsinki.

### 2.2 Experimental design

This pilot study was designed to compare the effects of RST with CRIs or IRIs on S_dec_, bLa, and WAnT, and it adopted a parallel-group design consisting of a total of 11 sessions. Nine sessions of RST were performed within 3 weeks and the frequency was two to four times per week, with at least one day of rest between RST sessions because cycling exercise primarily involves concentric contractions, which are less likely to cause muscle damage (10, 11). The weekly frequency was determined by taking the schedules of each participant and examiner into account. The WAnT was conducted before and after the RST. RST was performed across nine sessions within 3 weeks because eight to nine sessions are necessary to improve anaerobic work capacity (12–16). The participants were non-randomly assigned to either the CRI or IRI group based on their WAnT results and pre-intervention load, as power output during training influences physiological adaptations (17). The WAnT was used as an indicator of anaerobic work capacity in the pre- and post-training periods, owing to the high metabolic contribution of adenosine triphosphate, adenosine triphosphate-phosphocreatine (ATP-PCr), and the glycolytic system (18). The S_dec_ for all sessions was calculated as an index of muscle fatigue. The bLa concentration was measured after RST in the first and last sessions to assess the participants’ glycolytic response during training. Additionally, bLa was measured after the WAnT in the pre- and post-training periods to assess systemic adaptation. All the experiments were performed under the supervision of at least one examiner.

### 2.3 Participants

In total, 20 male Japanese participants initially took part in this study. However, three participants did not complete all the experiments due to personal reasons. Therefore, 17 participants who completed all the experiments, with eight in the CRI group (age: 23.8 ± 5.3 years; height: 173.0 ± 8.8 cm; body mass: 70.2 ± 8.7 kg) and nine in the IRI group (age: 25.1 ± 4.2 years; height: 173.6 ± 7.3 cm; body mass: 69.1 ± 11.2 kg), were included in the analysis. Moreover, 15 participants did not participate in daily sports. One participant in the CRI group played basketball once per week, and one participant in the IRI group played soccer three times per week. However, these sports activities were not restricted during the intervention period. The participants were instructed to refrain from performing strenuous exercise that could induce muscle damage on the day before and the day of each experiment. Moreover, the participants were prohibited from performing resistance training of their lower extremities and endurance training but they were instructed to maintain their current eating habits during the intervention period.

### 2.4 RST

**Figure 1:**
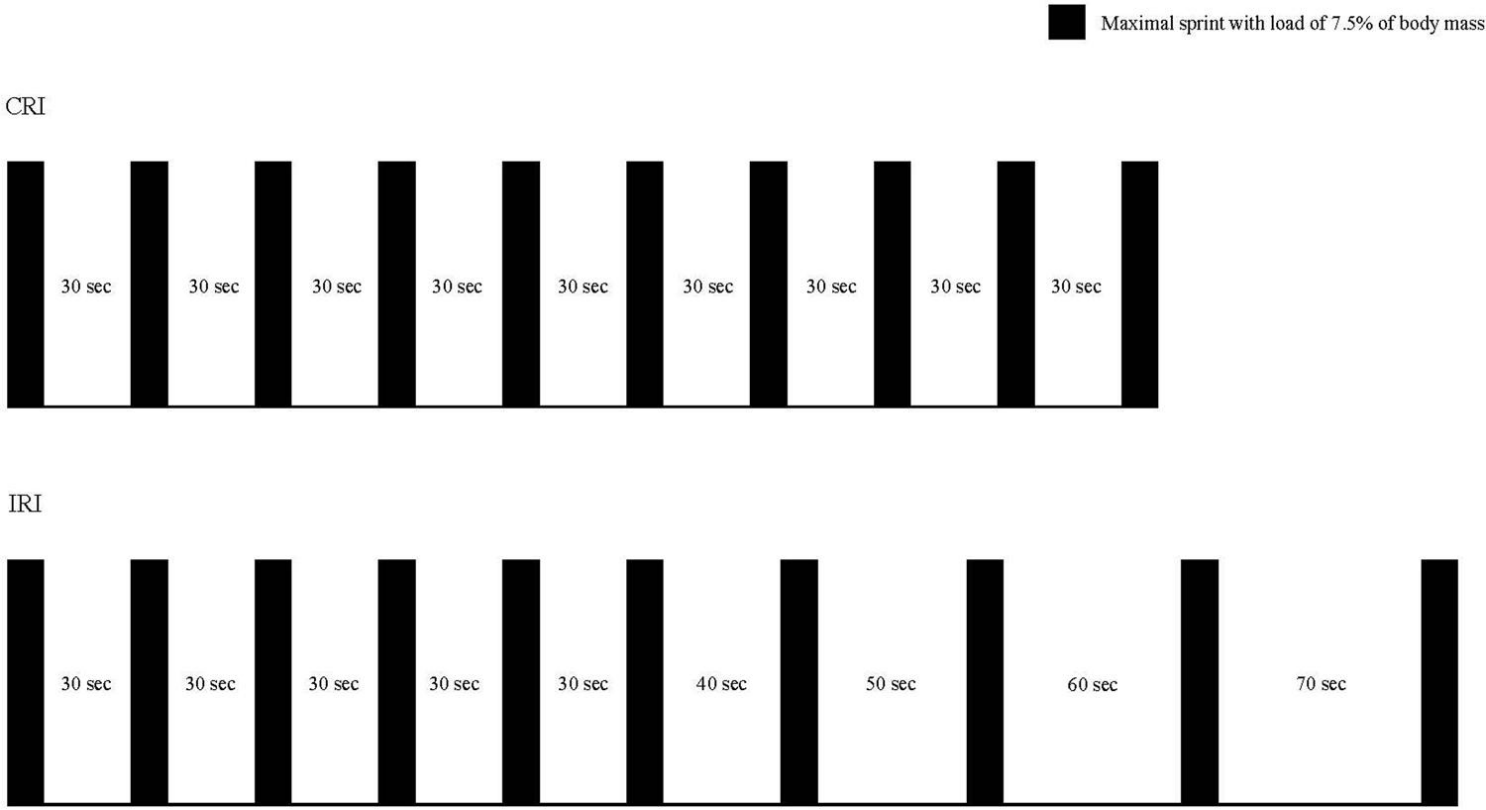
Repeated sprint training protocols of CRI and IRI. CRI, constant rest interval; IRI, incremental rest interval.

In both groups, the RST consisted of 10 sets of 10-s maximal sprints using a cycle ergometer (Fujin-Raijin, OCL Co., Ltd., Tokyo, Japan) with a load of 7.5% of the body mass of the participant. The load and sprint duration were similar to those reported by Kavaliauskas et al. (13). However, the number of sets performed was increased to 10, as six sets did not improve WAnT performance (13). The CRI was set as a 30-s rest interval between sets throughout RST, whereas the IRI was set as a 30-s rest interval from the first to the fifth set, and the rest interval was increased by 10 s from the sixth set onward (Figure 1). The first reason for the gradual increase in the rest interval by 10-s after the sixth set was based on findings from a previous study by Billaut and Basset (7), in which repeated sprint exercise with 5-s rest increments had no positive effect on the total work volume. Moreover, the second reason was that it was necessary to keep the work-to-rest ratio within 1:7 because a work-to-rest ratio of 1:8 or more has been classified as sprint interval training in many previous studies (19–21). The load remained constant throughout the training period in both groups. Prior to RST, all the participants first performed a warm-up cycling exercise (2.0 kp × 60 rpm) for 10 min. Thereafter, they rested for 1 min and performed maximal pedaling for 5 s to familiarize themselves with maximal pedaling using a load equal to that in RST. The RST was started with maximal pedaling for 5 s, followed by a 2-min-and-55-s rest. During the RST, the participants’ power was recorded every 0.1 s, and the total work volume of each session was calculated using the following formula:

$$\mathrm{Total}\;\mathrm{work}\;\mathrm{volume}\;\mathrm{of}\;\mathrm{each}\;\mathrm{session}\;(\mathrm{kJ})=\left\{\frac{\mathrm{mean}\;\mathrm{power}\;\mathrm{of}\;\;1\;\mathrm{set}\times 10}{1000}\right\}+\cdots +\left\{\frac{\mathrm{mean}\;\mathrm{power}\;\mathrm{of}\;\;10\;\mathrm{set}\times 10}{1000}\right\}$$

The total work volume of each session was summed, and the total work volume throughout the training period was calculated for both groups. Moreover, S_dec_ was calculated as a measure of muscle fatigue for each session using the following formula (2):

$$S_{\mathrm{dec}}\;(\text{\%})=\left\{1-\frac{\left(\mathrm{sum}\;\mathrm{of}\;\mathrm{peak}\;\mathrm{power}\;\mathrm{from}\;\mathrm{set}\;\;1\;\mathrm{to}\;\;10\;\mathrm{during}\;\mathrm{RST}\right)}{\mathrm{best}\;\mathrm{peak}\;\mathrm{power}\;\mathrm{during}\;\mathrm{RST}\times 10}\right\}\times 100$$

### 2.5 bLa

Blood samples were collected from participants' earlobes (0.3 μL), and bLa was measured using a bLa analyzer (Lactate Pro 2, Arkray, Kyoto, Japan). bLa reaches its peak value 3–8 min after exercise (22). Therefore, bLa was measured once immediately and 5 min after the RST and WAnT. One participant in the CRI group was excluded from the analysis at 5 min after RST because their bLa was not measured in time.

### 2.6 WAnT

The WAnT involved maximal pedaling for 30 s using a cycle ergometer (Fujin-Raijin, OCL Co., Ltd, Tokyo, Japan), wherein the load was set at 7.5% of the body mass of the participant. To minimize fatigue and circadian rhythm effects, the post-training measurements were performed after at least 2 days of rest since the last RST session, and the difference in the time of day between the pre- and post-training measurements was within 3 h. Notably, the range of days between the last RST session and the post-training measurement was 2–4 days in the CRI group and 2–5 days in the IRI group, and the Mann–Whitney *U*-test revealed no statistically significant difference between the groups (*p* = 0.481). The warm-up before the WAnT was the same as for RST. The examiner encouraged the participants during their maximal pedaling. Power was recorded every 0.1 s, and the participants’ relative peak and mean power values per body mass were calculated. The participants’ mean power every 5 s during the WAnT was also calculated.

### 2.7 Statistical analyses

The Shapiro–Wilk test was performed prior to the statistical analyses to confirm the normality of all data. When normality was observed for the data within and between groups, the unpaired *t*-test or two-way mixed-design analysis of variance (ANOVA) was performed as a parametric test. If an interaction was observed in the ANOVA, a *post hoc* analysis was performed using the Bonferroni test. If data were included for which normality was not observed within or between groups, the paired *t*-test or Wilcoxon signed-rank test was performed for within-group comparisons and an unpaired *t*-test or Mann–Whitney *U*-test for between-group comparisons. When paired or unpaired *t*-tests were performed, Cohen′s d was calculated as an index of the effect size and the 95% confidence interval (CI) of the mean difference was also calculated. Moreover, as indices of the effect size, partial *η*^2^ was used for the ANOVA and r was used for the Wilcoxon signed-rank test and Mann–Whitney *U*-test. All data are expressed as the mean ± standard deviation, and the significance level was set at *p* < 0.05. SPSS Statistics 28.0.1 software (IBM Corporation, Armonk, NY, USA) was used for all the statistical analyses.

## 3 Results

No statistically significant differences were observed in WAnT performance and load between the groups at baseline (figure not shown).

**Figure 2:**
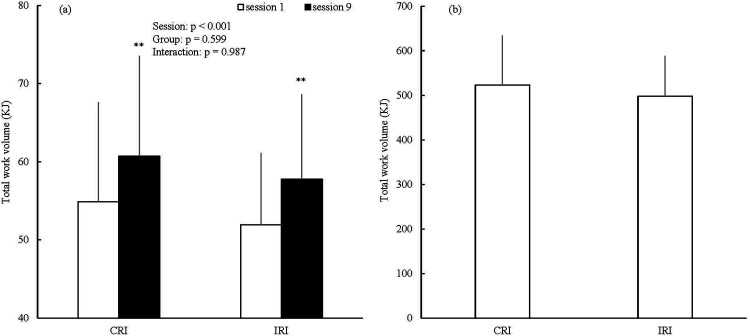
Results of total work volume in session one and session nine **(a)** and throughout the training period **(b).** CRI, constant rest interval; IRI, incremental rest interval; **p < 0.001 vs. session one; data are expressed as mean ± SD.

In the two-way (session × group) ANOVA results for total work volume in sessions 1 and 9, a significant main effect of session (partial *η*^2^ = 0.725; Figure 2a) was observed. However, no significant main effects were observed for group and the interaction (main effect of group: partial *η*^2^ = 0.019; interaction: partial *η*^2^ = 0.000; Figure 2a). No statistically significant difference was observed in the total work volume between the groups throughout the training period (unpaired *t*-test: *p* = 0.616; Cohen's d = 0.249; 95% CI of the mean difference = −79.247–129.321; Figure 2b).

**Figure 3:**
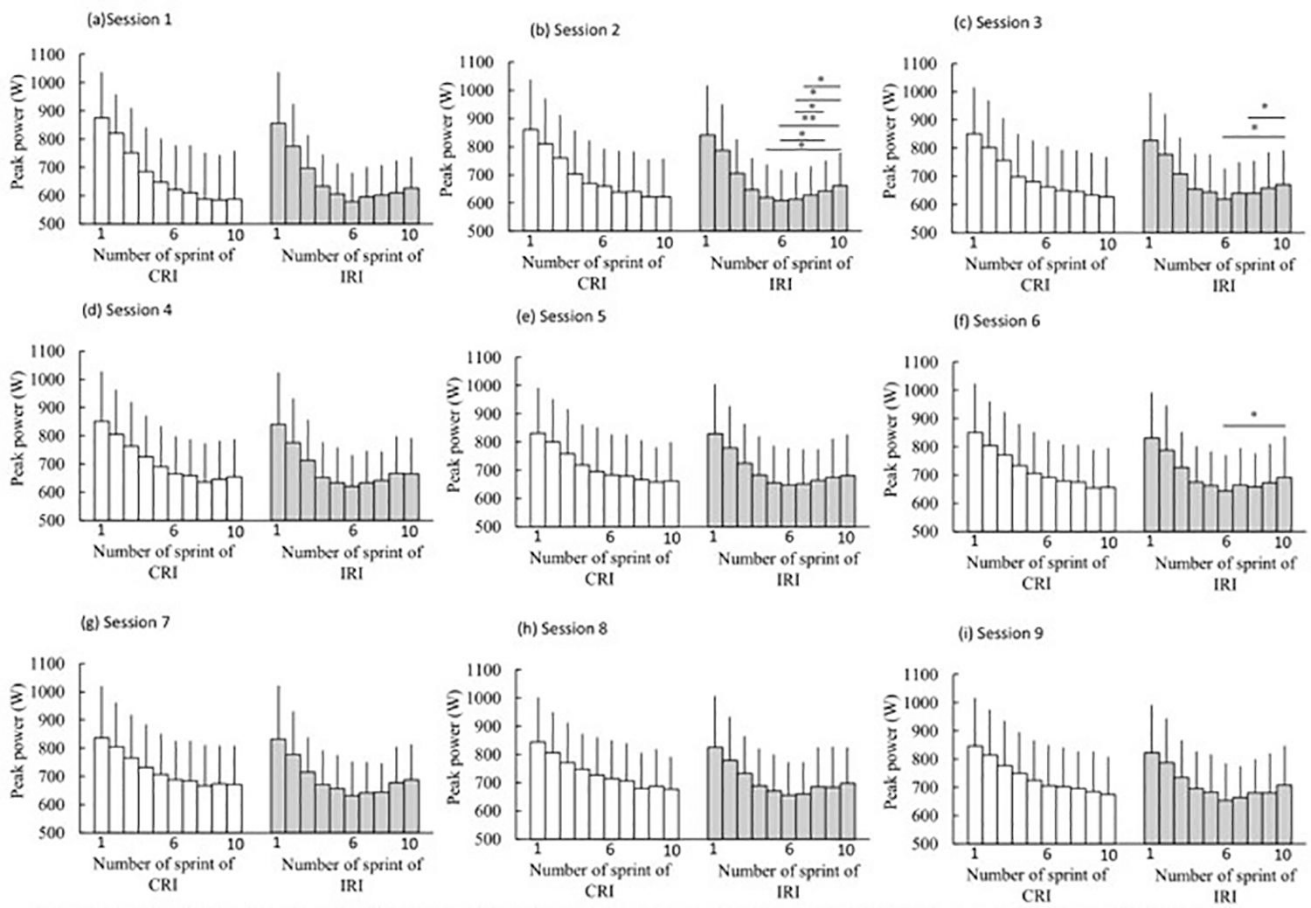
Results of peak power in each set during repeated sprint cycling training across all the sessions. Session 1 **(a)**, Session 2 **(b)**, Session 3 **(c)**, Session 4 **(d)**, Session 5 **(e)**, Session 6 **(f)**, Session 7 **(g)**, Session 8 **(h)**, Session 9 **(i)**. CRI, constant rest interval; IRI, incremental rest interval; data are expressed as mean ± SD.*P < 0.05, **P < 0.01.

Figure 3 shows the peak power results for each set during the RST across all sessions. In the two-way (set × group) ANOVA results, interactions were observed in some sessions, and the *post hoc* analyses indicated a recovery of peak power in the latter part of the RST in the IRI group (*p* < 0.05). However, no significant differences between the groups (*p* > 0.05) were observed. Moreover, in many sessions, no significant interactions were observed, and when interactions were observed, no significant differences in peak power between sets were detected in the *post hoc* tests (*p* > 0.05).

**Figure 4:**
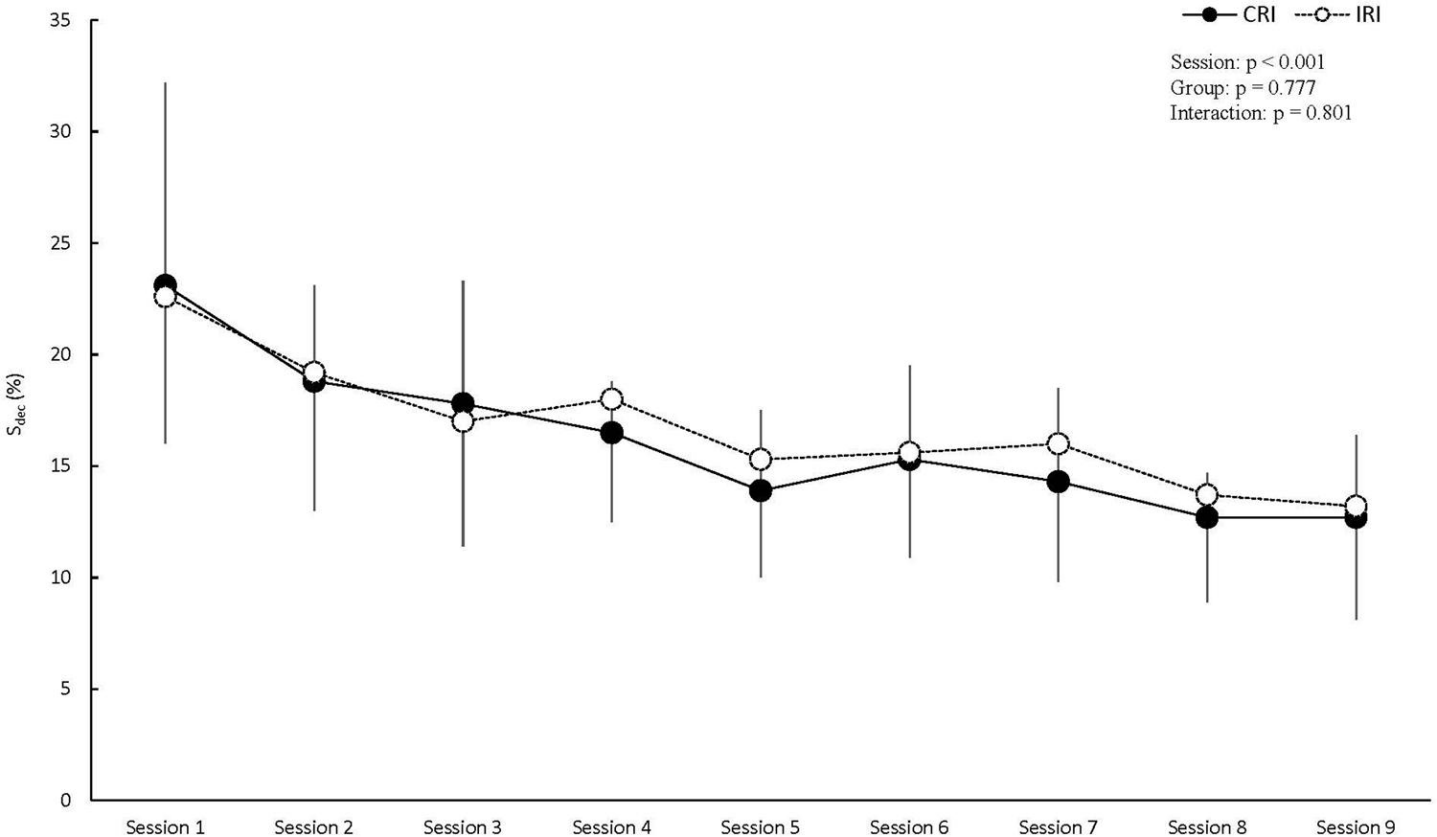
Decrement score results across all sessions. CRI, constant rest interval; IRI, incremental rest interval; S_dec_, decrement score; data are expressed as mean ± SD.

Figure 4 shows the S_dec_ results in all the sessions. The results of the two-way (session × group) ANOVA showed a significant main effect of session (partial *η*^2^ = 0.566). However, no significant main effects were observed for group or the interaction (main effect of group: partial *η*^2^ = 0.006; interaction: partial *η*^2^ = 0.025).

**Figure 5:**
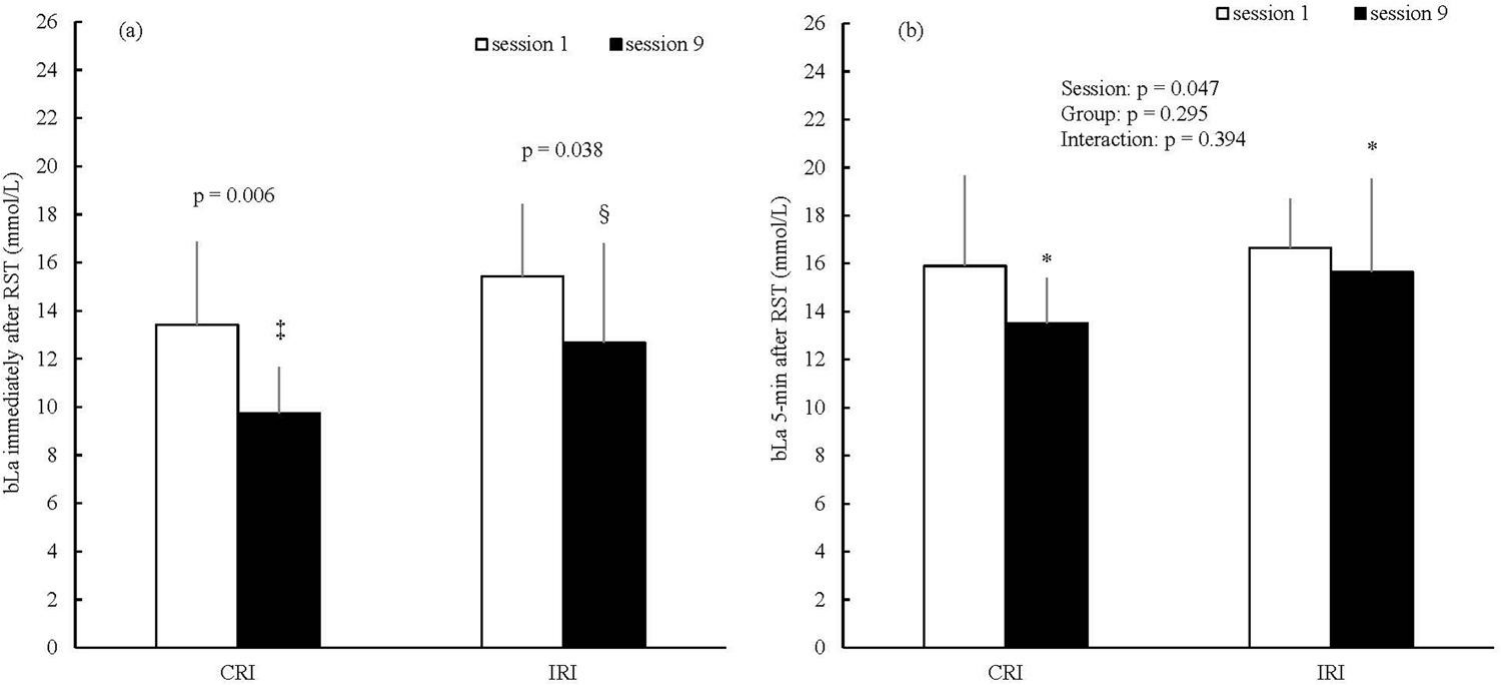
The bLa results immediately **(a)** and 5-min **(b)** after the RST. ‡*p* < 0.01 vs. session one in the CRI group; §*p* < 0.05 vs. session one in the IRI group; *p < 0.05 significant main effect of the session. Data are expressed as mean ± SD. bLa, blood lactate; RST, repeated sprint training; CRI, constant rest interval; IRI, incremental rest interval.

Figures 5a,b show the bLa results immediately and 5 min after the RST in sessions one and nine. The bLa immediately after RST was not significantly different between the CRI and IRI groups in session one (Figure 5a; unpaired *t*-test: *p* = 0.219; Cohen's d = −0.623; 95% CI of the mean difference = −5.351–1.331). The bLa immediately after RST was not significantly different between the CRI and IRI groups in session nine (Figure 5a, Mann–Whitney *U*-test: *p* = 0.200, r = 0.327). The bLa immediately after RST with CRIs was significantly decreased in session nine compared with that after session one (Figure 5a; paired *t*-test: Cohen's d = 1.149; 95% CI of the mean difference = 1.457–5.868). The bLa immediately after RST with IRIs was significantly decreased in session nine compared with that after session one (Figure 5a; Wilcoxon signed-rank test: *r* = 0.692). In the result of the two-way (session × group) ANOVA, a significant main effect of session was noted on the bLa 5 min after RST (Figure 5b; main effect of session: partial *η*^2^ = 0.252). However, no significant main effects of group and the interaction (Figure 5b; main effect of group: partial *η*^2^ = 0.078; interaction: partial *η*^2^ = 0.052) were observed.

Table 1 shows the results for body mass, load during the WAnT, peak and mean power during the WAnT, and bLa immediately and 5 min after the WAnT, both before and after the training period. In the results of the two-way (time × group) ANOVA, no main effect of time, group, or the interaction was observed on body mass, load during the WAnT, or peak power during the WAnT. Although a significant main effect of time was recorded in mean power throughout the WAnT, no significant main effect of group or the interaction was observed. A significant main effect of group was noted both immediately and 5 min after the WAnT on the bLa. However, no significant main effect of time or the interaction was noted immediately or 5 min after the WAnT on bLa.

**Table 1:** The body mass, load, and performance of the participants during the WAnT and their bLa after the WAnT.

Measurement	Group	Pre-training	Post-training	*p*-Value of the main effect of time (partial *η*^2^)	*p*-Value of the main effect of group (partial *η*^2^)	*p*-Value of the interaction (partial *η*^2^)
Body mass (kg)	CRI	70.2 ± 8.7	70.1 ± 8.3	0.535 (0.026)	0.866 (0.002)	0.283 (0.076)
	IRI	69.1 ± 11.2	69.5 ± 10.9			
Load during the WAnT (kp)	CRI	5.3 ± 0.7	5.3 ± 0.6	0.236 (0.092)	0.916 (0.001)	0.236 (0.092)
	IRI	5.2 ± 0.8	5.2 ± 0.8			
Peak power during the WAnT (W/kg)	CRI	12.4 ± 1.0	12.4 ± 1.3	0.488 (0.033)	0.673 (0.012)	0.690 (0.011)
	IRI	12.2 ± 0.9	12.1 ± 1.0			
Mean power during the WAnT (W/kg)	CRI	8.8 ± 0.5	9.2 ± 0.7	0.002 (0.494)**	0.283 (0.076)	0.439 (0.040)
	IRI	8.6 ± 0.7	8.8 ± 0.7			
bLa immediately after the WAnT (mmol·L^−1^)	CRI	5.0 ± 2.0	5.1 ± 1.5	0.209 (0.103)	0.037 (0.260)*	0.263 (0.083)
	IRI	6.5 ± 2.2	7.9 ± 3.0			
bLa 5-min after WAnT (mmol·L^−1^)	CRI	11.8 ± 2.3	11.7 ± 2.2	0.504 (0.030)	0.046 (0.240)*	0.481 (0.034)
	IRI	13.5 ± 3.2	14.4 ± 2.0			

WAnT, Wingate anaerobic test; bLa, blood lactate; CRI, constant rest interval; IRI, incremental rest interval.

Data are expressed as mean ± standard deviation.

* *p* < 0.05 significant main effect of the group.

** *p* < 0.01 significant main effect of time.

**Figure 6:**
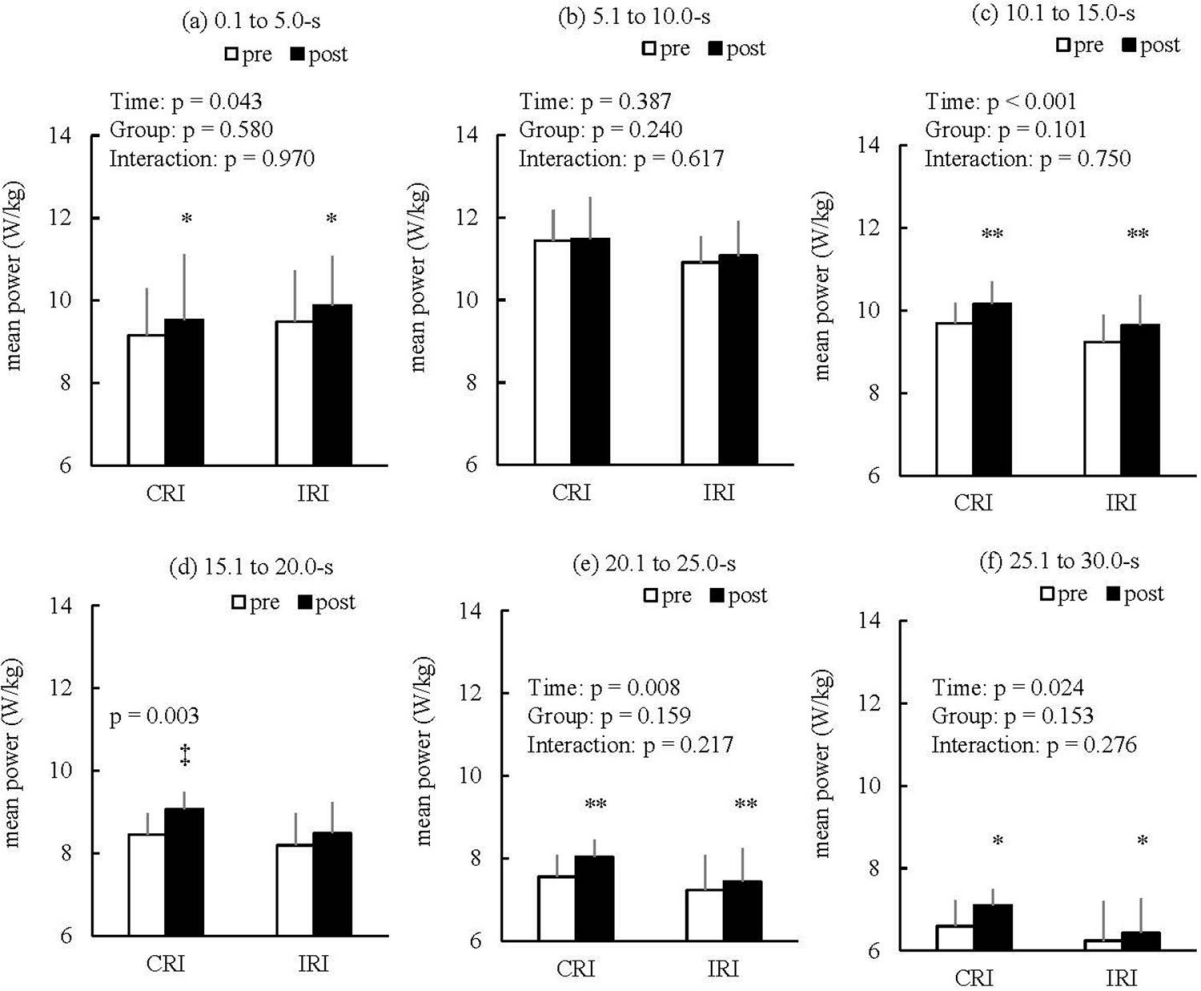
Results of the mean power at 0.1–5.0-s **(a)**, 5.1–10.0-s **(b)**, 10.1–15.0-s **(c)**, 15.1–20.0-s **(d)**, 20.1–25.0-s **(e)**, and 25.1–30.0-s **(f)** during the WAnT. ‡*p* < 0.01 vs. pre-training in the CRI group; **p < 0.01 significant main effect of time; *p < 0.05 significant main effect of time. Data are expressed as mean ± SD. CRI, constant rest interval; IRI, incremental rest interval; WAnT, Wingate anaerobic test.

Figures 6a–f show the mean power every 5 s during the WAnT. In the results of the two-way (time × group) ANOVA for the mean power at 0.1–5.0 s during the WAnT, although a significant main effect of time was observed, no significant main effect of group or the interaction (Figure 6a; main effect of time: partial *η*^2^ = 0.245; main effect of group: partial *η*^2^ = 0.021; interaction: partial *η*^2^ = 0.000) was noted. For the mean power at 5.1–10.0 s during the WAnT, no significant main effect of time, group, or the interaction (Figure 6b; main effect of time: partial *η*^2^ = 0.050; main effect of group: partial *η*^2^ = 0.091; interaction: partial *η*^2^ = 0.017) was observed. For the mean power at 10.1–15.0 s during the WAnT, although a significant main effect of time was observed, no significant main effect of group or the interaction (Figure 6c; main effect of time: partial *η*^2^ = 0.554; main effect of group: partial *η*^2^ = 0.169; interaction: partial *η*^2^ = 0.007) was noted. The mean power at 15.1–20.0 s during the WAnT increased from pre- to post-training in the CRI group (Figure 6d; paired *t*-test: Cohen's d = 1.269; 95% CI of the mean difference = 0.290–0.929). However, the mean power at 15.1–20.0 s during the WAnT did not increase from pre- to post-training in the IRI group (Figure 6d; Wilcoxon signed-rank test: *p* = 0.051; *r* = 0.652). Additionally, no significant difference was observed between the CRI and IRI groups in the mean power at 15.1–20.0 s during the WAnT post-training (Figure 6d, Mann–Whitney *U*-test: *p* = 0.093, *r* = 0.420). In the results the two-way (time × group) ANOVA for the mean power at 20.1–25.0 s during the WAnT, although a significant main effect of time was noted, no significant main effect of group or the interaction (Figure 6e; main effect of time: partial *η*^2^ = 0.382; main effect of group: partial *η*^2^ = 0.128; interaction: partial *η*^2^ = 0.100) was observed. For the mean power at 25.1–30.0 s during the WAnT, a significant main effect of time was observed, but no significant main effect of group or the interaction (Figure 6f; main effect of time: partial *η*^2^ = 0.294; main effect of group: partial *η*^2^ = 0.131; interaction: partial *η*^2^ = 0.079) was observed.

Table 2 shows the percentage change in mean power throughout the WAnT and the mean power every 5 s during the WAnT. According to the unpaired *t*-test, the percentage change in mean power throughout the WAnT and the mean power of all phases during the WAnT were not significantly different.

**Table 2:** The percentage change in mean power during the WAnT.

Phase during the WAnT (s)	CRI group	IRI group	*p*-Value	Cohen's d	95% CI of mean difference
0.1–30	4.7 ± 3.5	3.3 ± 4.8	0.504	0.332	−2.967–5.772
0.1–5.0	3.7 ± 7.2	4.4 ± 8.2	0.851	−0.093	−8.720–7.283
5.1–10.0	0.3 ± 3.8	1.5 ± 5.0	0.577	−0.277	−5.898–3.409
10.1–15.0	5.0 ± 4.7	4.5 ± 4.4	0.818	0.114	−4.180–5.210
15.1–20.0	7.4 ± 4.9	3.7 ± 4.6	0.126	0.786	−1.179–8.621
20.1–25.0	6.8 ± 6.5	3.0 ± 6.6	0.249	0.583	−2.954–10.573
25.1–30.0	8.1 ± 7.8	3.7 ± 11.0	0.368	0.451	−5.654–14.363

Data are expressed as mean ± standard deviation. WAnT, Wingate anaerobic test; CRI, constant rest interval; IRI, incremental rest interval; CI, confidence interval.

## 4 Discussion

This is the first study to examine the effect of RST with IRIs without matching the total rest duration, and, as will be described below, our hypothesis was partially supported. Billaut and Basset (7) reported that repeated sprint exercise with IRIs significantly lowers the total work volume compared with repeated sprint exercise with CRIs. However, this repeated sprint exercise was performed under conditions in which the total rest duration was matched, with extremely short rest periods in the first half of the repeated sprint exercise. Therefore, the findings of our study are important to confirm the effectiveness of RST with IRIs. From the perspectives of the S_dec_ and WAnT results in this study and time efficiency, there may be limited benefits for strength and conditioning coaches or personal trainers to prescribe RST with IRIs to athletes or clients.

In several sessions in the IRI group, a recovery of peak power was observed in the latter part of the RST (Figures 3a–i). In previous studies, the contribution of ATP-PCr and the aerobic system was higher in the latter half than in the first half of a repeated sprint exercise (2, 9). Moreover, PCr recovery is related to power output in the latter half of a repeated sprint exercise (23). Therefore, in several sessions in the IRI group, the incremental increase in the rest interval duration may have allowed PCr to recover, resulting in a recovery of peak power in the latter half of the RST. However, in many sessions in the IRI group, no recovery of peak power was observed, and no statistically significant differences in S_dec_ were found between the groups (Figures 3a–i, Figure 4). Therefore, to induce a more pronounced recovery of peak power that would be sufficient to produce significant differences between the groups, a longer incremental increase in the rest interval duration may be required.

As hypothesized, no significant difference was observed in the increase in glycolytic metabolism between RST with CRIs or IRIs. There is no consensus on whether repeated sprint exercise with shorter rest intervals results in a higher bLa concentration, an indicator of glycolytic metabolism, than repeated sprint exercise with longer rest intervals (3, 4, 6). However, the contribution of glycolytic metabolism is lower in the latter half of repeated sprint exercise than in the first half (2, 9). In this study, no statistically significant differences were observed in the bLa between the groups in sessions one and nine (Figures 5a,b). Hence, it is speculated that even when the rest intervals were extended in the latter half of RST with IRIs, glycolytic metabolism was equally increased when compared with that of RST with CRIs. In future studies, it would be desirable to include more detailed examinations, such as blood or muscle pH and the activity of enzymes involved in glycolytic metabolism (e.g., phosphofructokinase), because blood lactate concentration is only an indirect marker of glycolytic activity.

Additionally, it is speculated that the improvement in the mean power during the WAnT in both groups was due to improved ATP-PCr and aerobic systems. In this study, while the total work volume during the RST increased from session one to session nine in both groups (Figure 2a), the bLa after the RST significantly decreased from sessions one to nine in both groups (Figures 5a,b). When monocarboxylate transporter 1 (MCT1), which is involved in the removal of lactate from the circulation, is increased by training, lactate levels decrease during the same exercise compared to before the training (24). MCT1 concentration correlates significantly with the citrate synthase activity involved in maximal oxygen uptake, which is an indicator of aerobic capacity (25, 26). Hence, it is possible that aerobic capacity was improved by the RST in both groups. Moreover, the mean power at 0.1–5.0, 20.1–25.0, and 25.1–30.0 s improved in both groups during the WAnT (Figures 6a,e–f). However, the bLa after the WAnT did not significantly change between the pre- and post-training periods in either group (Table 1). Although the calculated contribution to power, work, and oxygen uptake during WAnT of the glycolytic metabolism was highest during the WAnT, the contribution of the ATP-PCr system was higher in the first half, while the contribution of the aerobic system was gradually higher in the latter half (18). On the basis of these results, it is possible that the improvement in the mean power during the WAnT may have been due to the improvement of the ATP-PCr and aerobic systems. Furthermore, this improvement is practically significant as it may help enhance performance in sports that require both anaerobic and aerobic metabolisms, such as repeated sprint ability or the 400 m track event (2, 27).

It is possible that RST with IRIs results in lower gains in aerobic capacity than that with CRIs. The contribution of the aerobic metabolism gradually increases during the latter half of the WAnT (18). Ulupınar et al. (6) also reported that aerobic metabolism was higher in repeated sprint exercise with shorter rest periods than in repeated sprint exercise with longer rest intervals. Here, the mean power at 15.1–20.0 s during the WAnT only increased in the CRI group, and no significant change was observed in the IRI group. Although the percentage changes in mean power from 20.1 to 25.0 s and 25.1 to 30.0 s were not significantly different between the groups, those in the CRI group were approximately twice as high as those in the IRI group (Table 2). Thus, it is possible that the RST with IRIs did not improve aerobic capacity compared to the RST with CRIs.

This study had two limitations. First, the sample size of this study was small. Therefore, further studies should verify the effectiveness of RST with IRIs using larger sample sizes. Second, there was one participant in each of the CRI and IRI groups who engaged in sports activities. Further studies should include participants with similar activity levels outside the intervention.

In future studies, it is desirable to verify the following three points to clarify the advantages of RST with IRIs. First, it is necessary to investigate the effect of RST with CRIs or IRI on VO_2max_ because differences in these effects may have affected the results of this study. Second, the efficacy of RST with IRIs may differ depending on the participant's VO_2max_ level because previous studies have reported a correlation between PCr recovery and VO_2max_ (28). Hence, it is also essential to examine the efficacy of RST with IRIs in athletes with higher VO_2max_ levels using a larger sample size. Finally, it is also necessary to elucidate the physiological responses and adaptations using a different IRI method than that used in this study as the effect may vary depending on the way the rest interval is extended.

## 5 Conclusions

In this study, we examined the effects of RST with CRIs or IRIs on S_dec_, bLa, and WAnT performance. S_dec_, as an index of muscle fatigue, showed no statistically significant differences between the groups in all sessions, indicating that muscle fatigue was similarly induced by both CRIs and IRIs during the RST. Similarly, bLa did not differ significantly between the groups in sessions one and nine. However, despite the increase in total work volume during RST from session one to session nine in both groups, the bLa was significantly decreased in both groups in session nine compared with session one. Therefore, it is possible that metabolic adaptations associated with lactate occurred in both groups. After performing sessions one to nine within 3 weeks, the participants’ peak power during the WAnT did not increase in either group. In contrast, the mean power during the WAnT increased significantly in both groups from pre- to post-training, but there were no significant differences between the groups. These improvements in mean power may reflect an increased power sustainability rather than peak explosiveness. Additionally, the mean power every 5 s during the WAnT increased significantly in both groups in the early (0.1–5.0 s), middle (10.1–15.0 s), and final phases (20.1–25.0 s and 25.1–30.0 s), but no significant differences were observed between the groups. The mean power at 15.1–20 s during the WAnT only significantly increased in the CRI group. Finally, the bLa after the WAnT did not significantly change in either group from pre- to post-training. Based on the power results during the WAnT and the post-WAnT bLa results, it is possible that anaerobic glycolysis did not change significantly in either group, whereas aerobic capacity may have improved in the CRI group. These results suggest that RST with IRIs may not provide additional benefits over RST with CRIs in untrained young adults.

## Data Availability

The raw data supporting the conclusions of this article will be made available by the authors, without undue reservation.
